# Constructing Complexity in a Young Sign Language

**DOI:** 10.3389/fpsyg.2018.02202

**Published:** 2018-12-13

**Authors:** Svetlana Dachkovsky, Rose Stamp, Wendy Sandler

**Affiliations:** Sign Language Research Laboratory, University of Haifa, Haifa, Israel

**Keywords:** language complexity, language emergence, sign languages, discourse relations, gesture, use of body, compositionality

## Abstract

A universally acknowledged, core property of language is its complexity, at each level of structure – sounds, words, phrases, clauses, utterances, and higher levels of discourse. How does this complexity originate and develop in a language? We cannot fully answer this question from spoken languages, since they are all thousands of years old or descended from old languages. However, sign languages of deaf communities can arise at any time and provide empirical data for testing hypotheses related to the emergence of language complexity. An added advantage of the signed modality is a correspondence between visible physical articulations and linguistic structures, providing a more transparent view of linguistic complexity and its emergence (Sandler, 2012). These essential characteristics of sign languages allow us to address the issue of emerging complexity by documenting the use of the body for linguistic purposes. We look at three types of discourse relations of increasing complexity motivated by research on spoken languages – additive, symmetric, and asymmetric (Mann and Thompson, 1988; Sanders et al., 1992). Each relation type can connect units at two different levels: within propositions (simpler) and across propositions (more complex).^1^ We hypothesized that these relations provide a measure for charting the time course of emergence of complexity, from simplest to most complex, in a new sign language. We test this hypothesis on Israeli Sign Language (ISL), a young language, some of whose earliest users are still available for recording. Taking advantage of the unique relation in sign languages between bodily articulations and linguistic form, we study fifteen ISL signers from three generations, and demonstrate that the predictions indeed hold. We also find that younger signers tend to converge on more systematic marking of relations, that they use fewer articulators for a given linguistic function than older signers, and that the form of articulations becomes reduced, as the language matures. Mapping discourse relations to the bodily expression of linguistic components across age groups reveals how simpler, less constrained, and more gesture-like expressions, become language.

## Introduction

The form of language is complex at each level of structure – the word, the phrase, the clause, and higher units in the linguistic hierarchy. And at each level, language is compositional – building up complex structures by combining and recombining simpler meaningful units. Children inevitably acquire this complex system, but not all at once. The gradual, step-by-step process of acquisition offers insight into the relative complexity of different language structures and their interaction (Brown, 1973; Barrett, 2016; Dromi, 2016; Tomasello and Brooks, 2016). The contribution of the child’s mind to this process is clearly impressive, but the process always occurs in the presence of adult models, which also contribute to acquisition. How does a new language accrue linguistic complexity from scratch? What are the characteristics of language emergence *de novo* in a community? Sign languages offer an opportunity to watch this phenomenon unfold.

This opportunity is unique to sign language in two ways: (1) they are young languages, and new ones can arise at any time and (2) there is often a direct correspondence between visible physical articulations and linguistic structures, providing a more transparent view of the emergence of complexity. The emergence of complexity with no model in spoken languages cannot be traced, because spoken languages are all thousands of years old or descended from old languages with complete linguistic structures. However, sign languages arise spontaneously in a community of signers, and if linguists are in the right place at the right time, they can observe the emergence of language.

It is generally assumed that sign languages begin life as gestural systems (Janzen and Shaffer, 2002; Goldin-Meadow, 2003; Meir et al., 2016) and interact with gesture even as they transform into linguistic systems (Liddell, 2003; Padden et al., 2013). Gestures are used by all of us, and they accompany spoken language. However, they are unlike language because they are less conventionalized and less systematic (McNeill, 1992). Established sign languages are clearly fully linguistic systems (Sandler and Lillo-Martin, 2006; Pfau et al., 2012). But how did they get that way?

A good deal of research has described the home sign system created by deaf children without a language model (Goldin-Meadow, 2003), and compared it with established sign language or with spoken language. Though most studies of the gestures that accompany speech are restricted to manual gestures, it has long been noted in studies of co-speech gesture that actions of the whole body accompany linguistic interaction (Kendon, 1972; Kidwell, 2013). But no study to date has attempted to combine measures of discourse complexity and bodily systematicity in order to map the emergence of language in a community, as we do here.

As will become clear, unlike the relation between form and meaning in spoken languages, many linguistic structures in sign languages have overt physical form, so that actions of different articulators – the hands, face, head, and torso – convey particular linguistic information. This is another advantage for our pursuit, as we explain. We do not claim that all aspects of sign language structure have overt physical correlates. We accept, for example, that syntactic, semantic, and other relations and properties can be covert, and that evidence for them can be attained through linguistic analysis of the same kind that applies to spoken language (Sandler and Lillo-Martin, 2006; Pfau et al., 2012). However, the unique kind of direct mapping that we will demonstrate offers an opportunity to observe particular linguistic properties directly as they unfold.

One study to date has adopted the strategy of matching bodily form to linguistic function in a newly emerging language, Al Sayyid Bedouin Sign Language (ABSL). This language was formed in a Bedouin village in present day Israel, after four deaf children were born in a single household, and deafness began to proliferate throughout the village (Sandler et al., 2005). The study showed that with each new generation of signers, additional articulators were recruited to convey increasing linguistic complexity as the language developed (Sandler et al., 2011; Sandler, 2012). In particular, Sandler (2016) found that the first overt markings to appear served to organize discourse functions, such as topic-comment structure, referent perspective, and topic continuity across a discourse – and in that order. This approach and its preliminary findings motivate the current study, and we describe it in more detail in **Emerging Sign Languages: Use of Body Articulators**.

We investigate the emergence of complexity in a different sign language of the same age as ABSL, Israeli Sign Language (ISL). This language has developed under different social conditions from those of ABSL (described briefly in **The ISL Community and the Formation of the Language**), leading to certain differences in the path of emergence (Meir et al., 2012; Meir and Sandler, in press). In more than a decade of research on ABSL, the research team refrained from attributing complex syntactic structure to utterances without overt evidence, and instead based their analyses primarily on the meaning and prosodic structure of the productions, and their interaction (Padden et al., 2010b; Sandler et al., 2014, 2005). We follow that strategy in the present study of ISL as well.

We adopt the theory of discourse relations proposed by Mann and Thompson’s (1988) and Sanders et al.’s (1992), laid out in **Measuring Relations and Complexity in Discourse**, to investigate the degree of complexity of utterances, and to measure the frequency and systematicity of the discourse relations they convey, including, for example, dependency between clauses. We look at three types of relations of increasing complexity, motivated by research on spoken languages. In the spirit of Mann and Thompson (1988) and Sanders et al. (1992, 1993), we adopt the following terms: additive, symmetric and asymmetric (see **Types and Levels of Discourse Relations and Their Relative Complexity**). Each of these types of relations can occur at two different levels: within propositions (simpler) and across propositions (more complex). We hypothesized that these constructions provide a measure for charting the emergence of complexity in a young language, from simplest to most complex, and tested the hypothesis on ISL.

By studying the recruitment of articulators to express linguistic form in fifteen ISL signers from three generations, we show in the section **Discussion: Bodily Marking Emerges Gradually** that this is indeed the case. In other words, the emergence of constructions reflects the degree of complexity in terms of relation type and level. We also find evidence for increasing systematicity and automaticity of form as the language matures. Our conclusions and suggestions for future research comprise the **Conclusion**.

### The ISL Community and the Formation of the Language

Israeli Sign Language is the established language of the deaf community in Israel (Meir and Sandler, 2008). It is a young sign language, roughly about 90 years old, which arose with the formation of the deaf community in Israel around the 1930s, beginning with the establishment of the first Israeli School for the Deaf in 1932 in Jerusalem. Immigrants from all over the world contributed to the signing used by a small number of deaf Jews and Arabs already in Jerusalem. Vocabulary items have been traced to a small number of immigrants from Germany, and immigrants from elsewhere in Europe, North Africa, and the Middle East also brought their sign languages or home sign systems with them. A conventional local sign language evolved, and today, ISL is used in a wide range of settings including the educational system, deaf social and cultural institutions, interpreting programs, and the media.

The linguistic structure of ISL is investigated in earlier work (e.g., Meir, 1998, 2010; Meir and Sandler, 2008; Meir and Sandler, in press) and its emergence has recently become the object of study, briefly noted in **The Body as a Marker of Linguistic Complexity** below.

### The Body as a Marker of Linguistic Complexity

Signers exploit the use of the hands, torso, head and facial expression to convey linguistic information. Early sign language research demonstrated that non-manual signals play important roles in American Sign Language (ASL) grammar by systematically co-occurring with various linguistic structures: questions, topics, conditionals, and others (e.g., Baker and Padden, 1978; Liddell, 1978, 1980; Baker-Shenk, 1983; Reilly et al., 1990). Later, similar phenomena were demonstrated in other sign languages (Bergman, 1984, Swedish Sign Language; Engberg-Pedersen, 1990, Danish Sign Language; Coerts, 1992, Sign Language of the Netherlands (NGT); Nespor and Sandler, 1999, Israeli Sign Language; Sutton-Spence and Woll, 1999, British Sign Language; Herrmann and Steinbach, 2013, German Sign Language). Although most of this research studied facial expressions, a few studies focused on the role of other articulators, such as the head (Sandler et al., 2011; Dachkovsky et al., 2013; Lackner, 2013; Puupponen et al., 2015) and torso (Wilbur and Patschke, 1998; Ormel and Crasborn, 2012); also see Pfau and Quer (2010) and Sandler (2010) for overviews of non-manuals in sign languages.

**FIGURE 1:**
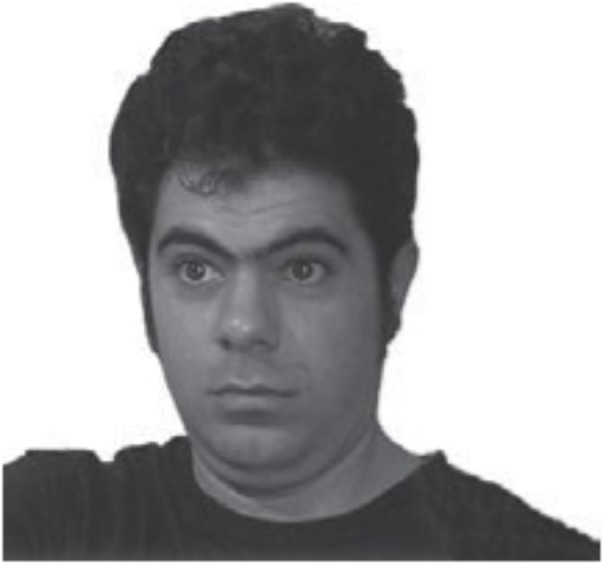
Typical intonational display of the antecedent clause of an ISL neutral conditional in a sentence meaning, *“If you eat now, you won’t be hungry for lunch.”* The image was captured while the signer was producing the underlined sign.

While early work on the role of non-manual markers in various structures such as interrogatives, topics, and relative clauses attributed them to the syntactic level of analysis (e.g., Liddell, 1978, 1980; Neidle et al., 2000), other researchers have argued that facial expressions and head movements are driven by various information structure and discourse considerations, such as topic continuity, foregrounding-backgrounding in subordinate constructions and others (Dachkovsky et al., 2013; Sandler et al., in press). For example, Figure 1 below illustrates the marking of a neutral conditional in ISL. It typically consists of raised eyebrows and forward movement of the head (Dachkovsky and Sandler, 2009).

**FIGURE 2:**
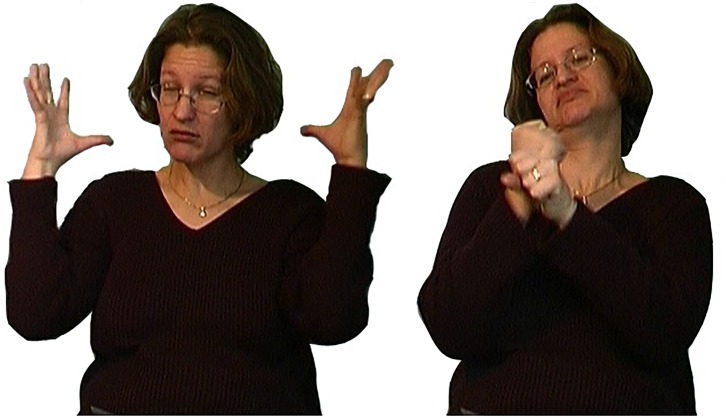
Typical intonational display of the antecedent clause of an ISL counterfactual conditional and its change in the consequence clause, in a sentence meaning, *“If the goalkeeper had caught the ball, the team would have won the game”* and glossed [GOALKEEPER HE CATCH-BALL] [WIN]. The figure shows the underlined signs.

Moreover, those signals can co-occur with other signals to create a more complex grammatical meaning. Thus, raised brows and forward head movement signaling conditionality can combine with squinted eyes to create a more complex linguistic form – together they systematically mark counterfactual conditionals in ISL (Dachkovsky, 2008). An example of this subordinate construction using a complex array of facial expressions and head movements is presented in Figure 2. The antecedent, or ‘if’ clause, in this example comprises an intonational phrase, and the head position and facial intonation align with the timing of the hands to mark the phrasal boundary. Thus, the head posture (head movement forward) and facial expression (raised brows and squinted eyes) change between the last sign of the first clause and the first sign of the second clause. This change follows the manual cue at the intonational phrase boundary – the hold in position of the last sign CATCH-BALL of the first clause.

These findings motivated a study of a newly emerging Bedouin sign language, which we discuss below. The study demonstrated that, in addition to the hands, head, and face, the torso and the non-dominant hand independently can be recruited for discourse organization. Since each articulator contributes additional linguistic information, recruitment of more articulators for different functions implies more complexity of language structure.

Taking the findings across sign languages into consideration, we arrive at a very general model that relates bodily articulators to linguistic roles across sign languages (see Sandler, 2018 for more details). This sort of correspondence is derived from a range of data and methods of analysis in different sign languages, and awaits statistical confirmation. Here our point of departure is that reliable correspondences between articulator activation and linguistic roles exist, and we test them statistically across different generations of signers in a young sign language.

### Emerging Sign Languages: Use of Body Articulators

The new field of emerging sign languages has laid the foundation for understanding how language arises. Yet, in most cases, these studies evaluate particular structures at the level of the word, the clause, or the sentence, without generalizing across levels. Since sign languages are transmitted in the visual modality and use multiple articulators, the findings of most earlier studies do note the role of the body in the process of the emergence of complex grammatical distinctions, but only indirectly.

Here we aim to overcome these limitations. First, the theory of discourse relations that we adopt allows us to make broader generalizations about the emergence of complexity across the clause and the sentence levels. Second, we exploit the direct relation between bodily action and linguistic structure to evaluate the emergence of complexity and systematicity.

Senghas (1995) and Senghas et al. (1997) were pioneers in this field. They have claimed rapid language development and change between cohorts of children in a deaf school in Nicaragua. In their work, the researchers focused on the development of temporal and spatial devices in this rapidly developing language (Senghas and Coppola, 2001; Kocab et al., 2016). Assignment to a cohort reflects both the age at which the signers arrived at the newly established school, and whether or not they had signing models in the environment. Members of the first cohort were older when they arrived at the school, and had no models for creating a language, while the second cohort were younger and had the advantage of the older cohort as a language model.

In their work, the researchers examined the emergence of particular discourse signals, often called referential shift devices – that is, devices, which shift the perspective of the discourse. In addition to lexical labels, sign languages can mark the shift with a manual point or with a movement of the body to a specified location in the three-dimensional space in front of the signer, capitalizing on the spatial affordances of the visual modality. While there was no significant difference between age cohorts of Nicaraguan Sign Language (NSL) in the use of neutral lexical signs and indexical points, there was a difference in the use of spatial devices (e.g., indexical points to space, body shifts and spatially modulated signs), with second-cohort signers using them significantly more (Kocab et al., 2015).

Ergin (2017) investigated the development of a recently discovered young sign language – Central Taurus Sign Language (CTSL), which emerged in the 1960s in a remote village situated in the mountainous region of Southern Turkey. The researcher reported on the emergence of a phonological system, handshape classifiers and argument structure in this village sign language, with a special focus on the way the semantic complexity in various different scenarios is realized on the surface structure of such a young language. In her study of the word order in this language, the author also demonstrated that the more specified use of body articulators (‘body segmentation’) in signaling reciprocal argument relations in a sentence is more characteristic of the younger signers’ production (Ergin, 2017).

The youngest reported sign language – the Sao Tome and Principe Sign Language (LGSTP) – started to emerge just a few years ago and is still in its first age cohort. The research group investigating this language conducted a longitudinal study through a few successive sessions of video recordings. One of their findings is that the earlier stages of language development are characterized by larger signing space than subsequent, later stages (Mineiro et al., 2017), measured according to the size of the joints involved in sign production.

By and large, all the studies mentioned so far have traced the emergence of the signed word, morphological complexity, and syntax within the sentence. As noted above, their findings related to the use of the body were an artifact of studying languages conveyed by corporeal articulators. Taking linguistic functions as the point of departure, their strategy can be summarized as a function-to-body approach.

A different perspective has arisen in the studies investigating a young sign language that emerged in a Bedouin village in the Negev desert of present day Israel. ABSL has been the object of study for over a decade by Aronoff et al. (2008) and Sandler et al. (2014, for an overview). ABSL began with four deaf children in a single family about 90 years ago, and the deaf population has since spread throughout the village, now numbering about 150 deaf people in a village of 4,000. ABSL has been developing across generations of signers. In their work on this young language, the team has been especially interested in the externalization by the body of the emergence and development of grammatical functions. The researchers paid special attention to manual timing and to use of the face and head in order to understand the structuring of sentences in ABSL (Sandler et al., 2005, 2011; Padden et al., 2010a).

Taking those studies as a basis, Sandler (2012) explicitly addressed the emergence of ABSL from a body-to-function perspective. With this approach, she investigated broader discourse functions, such as the discourse topic. This preliminary study traced the step-by-step recruitment of different articulators – the face, the head, the torso, and the non-dominant hand – to create an increasingly complex linguistic system in ABSL. In this way, a correlation was found between the increase in language complexity and the affordances of multiple bodily articulators participating in language expression at higher levels of discourse.

Two observations emerge from this work, which are relevant for the present study. The first is that there is often a direct correspondence between linguistic and bodily complexity. The second is that the body traces the order of emergence of linguistic structure, such that words on the hands are first; propositions and links between them, signaled by the head and face, are next; and broader discourse organization, embodied in movement of the torso and independent spatial placement of the non-dominant hand, is last. Details of this emergence are expanded in Sandler (2012, 2013).

Although ABSL studies rely on a small number of participants, due to the exigencies of fieldwork in a community of this kind, ISL offers a field that is much less limited, both in the size of the deaf population (estimated at about 10,000) and in their availability. At the same time, ISL arose under very different conditions, and can be considered a creole of many substrates but no superstrate (Meir and Sandler, 2008), so that the stages of its emergence may be less crisply defined. Nevertheless, concrete results about the emergence of this language have been reported. For example, conventionalization of the use of space was studied in Padden et al. (2010a). Another study found consistent and quantifiable relations between the increasing organization of bodily articulators and of linguistic structure in this language (Stamp and Sandler, 2016). Specifically, younger signers are more likely than older signers to use the head and the body simultaneously for separate linguistic functions.

In a study of relative clauses across age groups, Dachkovsky (2017) found that younger signers, unlike older signers, consistently organize this construction by aligning the noun and predicate of the relative clause with characteristic head positions and facial expressions. In the youngest group, the bodily markers are phonetically reduced, indicating the increased automaticity and conventionalization typical of grammaticalization (Dachkovsky, 2017). In general, these studies point to increased language complexity tied to increased articulatory complexity, as well as increased efficiency in use of different parts of the body as a language matures.

Here we develop the body-to-function approach and investigate the emergence of complexity and conventionalization in finer resolution. Specifically, we trace the relationship between the recruitment of bodily articulators and the complexity of discourse relations both within and across propositions. We adopt a particular measure of discourse relations and complexity, described in **Measuring Relations and Complexity in Discourse**, and investigate their emergence by tracking actions of the body for different linguistic units across age groups. We review relevant properties of language change, such as convergence as well as reduction of form in the process of conventionalization.

### Measuring Relations and Complexity in Discourse

We approach the study from the perspective of relations among constituents in a discourse, and their relative complexity. By discourse, we mean a coherent multi-utterance dialog or monolog text. Discourse contains propositions, where propositions are usually understood to be truth bearing statements denoting states of affairs (e.g., Wittgenstein, 1961; Krifka, 2001; Cristofaro, 2003), but it is more than a sequence of propositions. Despite some key differences, all definitions view discourse structure as the conceptual organization of a text, driven by the communicative goals of language users, the direction of information flow and considerations of common ground.

Discourse structure subsumes such notions as segmentation, anaphoric relations, and relations between segments (Kruijff-Korbayová and Steedman, 2003). As a result, stretches of discourse are analyzed as connected to each other through a range of discourse relations (see, among others, Gernsbacher and Givon, 1995; Graesser et al., 1997; Noordman and Vonk, 1997).

In the present study we are concerned only with relational aspects of discourse organization. Discourse relations usually connect events and situations described in propositions, and, therefore, cross the bounds of isolated propositions (cf. Hobbs, 1979; Mann and Thompson, 1986; Sanders et al., 1992, 1993). Yet, elements at a lower discourse level, within propositions, also contribute to discourse connectivity. For example, relations between topic and comment contribute substantially to information packaging.

In discourse, both explicit and implicit devices signify links between propositions, and between groups of propositions (e.g., Mann and Thompson, 1988; Kruijff-Korbayová and Steedman, 2003). Our approach encompasses both conceptual relations in discourse connectivity and overt linguistic signals. This approach is especially appropriate in the signed modality, where formal syntactic machinery for signaling these relations may be missing, controversial, and/or lacking empirical support, particularly in new sign languages.

Not all discourse relations contribute to complexity in the same way. In **Types and Levels of Discourse Relations and Their Relative Complexity** and **Increasing Complexity in Language Ontogeny, Diachrony, and Typology**, we introduce the types and levels of discourse organization that we adopt in terms of complexity, and briefly survey empirical evidence.

#### Types and Levels of Discourse Relations and Their Relative Complexity

A significant part of the discourse literature has focused on the question of how various sets of relations should be organized and what principles guide their groupings. Sanders (1997, 121) determined the properties common in all relations, in order to define *“the relations among the relations”* relying on the assumption that some discourse relations are more alike than others (see also Mann and Thompson, 1988). Within an organized system of discourse, its segments^2^ may bear relation to the system as a whole, or to each other, or to both. On these grounds, some discourse relations are described as more basic and others as more complex. We adopt here the general principles of an approach that distinguishes relations based on two criteria: *types* and *levels* (Sanders et al., 1992; Givón, 1995).

The first criterion, relation *type*, distinguishes between degrees of connection between the units of discourse, ranging from additive (weakly connected) to asymmetric (strongly connected) relations. In *additive*^3^ relations, basic units bear relations to the system as a whole but not to each other (Sanders et al., 1992). A more complex type of relation is *symmetrical* (e.g., Cristofaro, 2008; Givón, 2009a; Nir and Berman, 2010): units of the same rank are related both to the system as a whole, and to each other. Symmetric type relations can involve either coordinate (*e.g., I walk to work and also walk back home)* or contrastive relations *(e.g., I walk to work but drive back home).* In a third type of organization, known as *asymmetric* relations, the units are not of the same rank; one unit is dependent on another unit in the system (e.g., Cristofaro, 2008; Givón, 2009a; Langacker, 2009; Nir and Berman, 2010; see Table 1).

**Table 1:** Examples from our data for each relation.

	Within proposition	Across proposition
Additive	(1) My father was [hammering], [sawing], [and painting].	(4) [I talked with Dani], [Kate danced with Peter], [and Sveta ate her sandwich].
Symmetric	(2) [As well as sign language (being important)], [I don’t rule out the importance of spoken language].	(5) (The basketball game), it was [Tel Aviv] vs. [Haifa].
Asymmetric	(3) [My father and his wife] [they both had to stay].	(6) (Doctor said)… [because you’re not getting vitamin B12], [you need injections].

The units in example (1) in Table 1, show additive relations within a proposition, where the only relation between them is that they belong to the same whole. The order of items in *additive* relations does not change the meaning of the proposition. Example (2) is different because the units of the same rank are related *symmetrically* to each other through contrast. In (3), the elements of the utterance are not of the same rank so that the topic, ‘My father and his wife,’ serves as an anchor for the subsequent predication. We can see that they are related *asymmetrically*, since the former serves as a background for the latter, and their order cannot be reversed without changing the meaning of the utterance.

The second criterion relates to the level of language items across which the relations hold. Thus, the same major types of relations exemplified in 1–3 can also hold across propositions, exemplified in examples 4–6, resulting in more complexity within the same types of relations.

Asymmetrical relations across propositions are prototypically signaled by syntactically subordinate constructions, as in (6), where the asymmetric temporal relations between two propositions are manifested explicitly by the subordinating conjunction and different tense agreement in the English translation.

If categorization of discourse relations in terms of complexity is significant apart from purely descriptive considerations, it should prove relevant in areas such as language development, both synchronically (language acquisition) and diachronically (language change). In both areas, there is substantial supporting evidence for the increasing complexity of these relations.

#### Increasing Complexity in Language Ontogeny, Diachrony, and Typology

The increasing complexity of discourse relations is reflected in the order of development of its markers, both in ontogeny (language acquisition) and in diachrony (e.g., grammaticalization, pidgin and creole studies). Such general trends are reported in the works of Bloom (1973), Bowerman (1973), Bates (1976), Scollon (1976), Givón (1979, 1987, 2009a), Bickerton (1981), Bickerton (1990), Heine and Kuteva (2007), or Sanders et al. (2009), and others.

In stages of child language development, increasing complexity is well documented (Bloom, 1973; Bowerman, 1973; Scollon, 1976; Ochs and Schieffelin, 1979; Givón, 2009b). The focus has typically been on the use of connectives such as conjunctions reflecting age-related command of complex syntax (e.g., Jisa, 1987; McCabe and Peterson, 1991; Berman, 1996; Akinci and Jisa, 2000; Diessel, 2004), where syntactic development progresses from linear juxtaposition. Relations between clauses are first implicit (e.g., Hobbs, 1979; Diessel, 2004) then coordination is acquired, followed by subordination and embedding (Verhoeven et al., 2002). This progression corresponds to additive, symmetrical, and asymmetrical relations, within and across propositions in the Type and Level Approach adopted in this study.

Similarly, in historical change, in both established languages and in creole genesis, the development from additive relations to asymmetric relations across propositions has gained extensive empirical support (e.g., Heine and Kuteva, 2007; Hilpert and Koops, 2009; Koops and Hilpert, 2009). In addition, connective devices initially used at the level of the proposition often grammaticalize into various types of connectors across propositions. For example, the clause-final locative demonstrative *ia* ‘here,’ originally characterizing a location in a single proposition in Tok Pisin, developed to signal particular relations across propositions – between relative and main clauses (Sankoff and Brown, 1976).^4^

#### Conventionalization, Convergence, and Reduction

A critical part of language emergence and change is conventionalization. Through conventionalization, a language community converges on a consensus about the relationship between forms and their meanings. In a comparison of two young sign languages, ABSL and ISL, Meir and Sandler (in press) showed that language begins with extensive variation at all levels of structure, before gradually converging on conventionalized forms. They also showed that different parts of the grammar (e.g., phonology and different kinds of morphology) conventionalize at different paces, so that different aspects of linguistic organization should be evaluated in their own right, a direction that we take here.

In established languages, diachronic changes are sometimes driven by a tendency to distinguish grammatical meanings with distinctive forms, to make language more precise and, as a result, more explicit. For example, the Latin subordinating concessive conjunction *quamvis* started out as a clause-internal marker of speech-situation evocation meaning something like ‘as you want’ and grammaticized to a marker of concessive, adversative relations between propositions. As often happens in grammaticalization, at the beginning of this process, *quamvis* occurred interchangeably with other conjunctions, but gradually became the most frequent marker of the concessive/adversative relation in Latin, interpreted as ‘although,’ while the usage of other variants for this function decreased drastically. We will report a similar process of convergence and conventionalization in ISL.

In the process of conventionalization and specialization, the form of *quamvis* was also phonetically reduced – another measure of language emergence and conventionalization. The principle of economy (reduction of effort in the production of form) constantly interacts with the frequency of a language item, as demonstrated for example by Bybee (2010, 2013) in a series of studies on spoken languages. It is well known that the most frequent expressions tend to be reduced phonetically (see Zipf, 1935). In other words, information that is redundant because it is recoverable and/or predictable, either due to frequency of use or grammatical redundancy, tends to be reduced.

In sum, increase in complexity is accompanied by the development of linguistic signals *specialized* for a particular discourse function in a *systematic* (conventionalized) way, as well as by the *reduction of articulatory effort* (Lehmann, 2008; Bybee, 2010). It should be emphasized that language changes do not happen overnight; old forms do not give way to new without oscillation or variation. Changes occur gradually, as “orderly heterogeneity” is a fundamental characteristic of language (e.g., Weinreich et al., 1968; Labov, 1981). For example, in Labov’s (1981) discussion of the *a* split (i.e., tense/lax) in Philadelphia there is a steady movement from 0% tense *a* in the oldest speakers, a slight tendency toward tensing in speakers 40–60 years old, about 30% tensing among speakers in their twenties and thirties, and almost 50% among pre-adolescents and adolescents. Moreover, this growth in the tensing pattern does not occur evenly across the gamut of lexical items, but rather progresses incrementally by particular lexical items. Similar patterns of change have been observed for sign languages (Meir and Sandler, in press).

### Research Questions and Hypotheses of the Present Study

The present study elaborates and further supports the Type and Level Approach to understanding linguistic complexity and its emergence. We extend the approach to a language in a different physical modality from that of spoken language – a sign language. As ISL is a young language and some of its earliest users can still be recorded, we can track the development of complexity over time. We take advantage of the unique relation in sign languages between bodily articulations and linguistic forms, to investigate the accumulation of more complexity, more convergence in form, accompanied by reduction in this young language.

**FIGURE 3:**
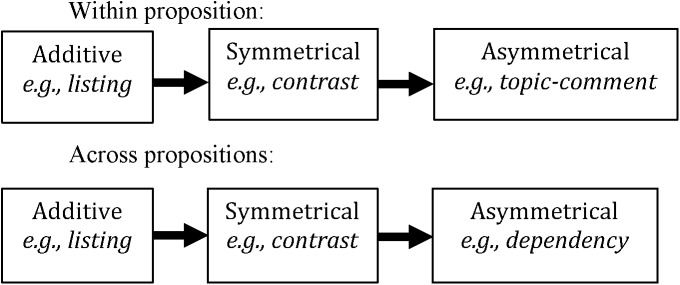
Schema of complexity cline.

We hypothesize that the Type and Level Approach predicts the course of emergence of linguistic complexity in ISL – from more basic additive relations to the more complex asymmetric relations, and from the lower, within-proposition discourse level to a higher, across-proposition level, as schematized in Figure 3 below.

In addition to increased complexity, we hypothesize that markings will become more systematic (i.e., more frequently used in the expected context), that signers will converge on fewer types of marking for a particular relation (like *quamvis* in Latin described above), and that their form will be reduced as the language matures.

## Methodology

This project follows the approach introduced in Sandler (2012), by analyzing language from the outside in, paying particular attention to the bodily articulators in sign languages and evaluating the linguistic structures that they manifest according to their meaning and discourse relations. Here we present evidence from ISL, a young sign language (about 90 years old) for the gradual emergence of linguistic complexity – from basic to complex language forms – through the gradual increase in systematicity and specificity of bodily signals.

To this end, we investigate the emergence of three relations, varying in terms of relation type (additive, symmetric, and asymmetric) and level (within or across propositions). We compare the frequency and systematicity of each relation produced by fifteen signers of different ages in 2 min each of spontaneous narrative. While spontaneous data are less controlled than elicited data, spontaneous data have the advantage of being natural and more ecologically sound than elicited data in terms of information structuring and the relations among constituents.

We adopt the apparent time hypothesis (Labov, 1963; Sankoff, 2006), which holds that language users do not change their language significantly after young adulthood, so that the language of older generations is a reflection of the state of the language in their youth. A comparison between older and younger signers enables us to make inferences about language emergence from the outset, since the language analyzed in this study is less than 100 years old.

According to the Type and Level Approach, we expect additive constructions to be present in the earliest stages of sign language emergence, as discussed in **Research Questions and Hypotheses of the Present Study**. Therefore, we expect signers of all ages to show similar frequency rates for additive constructions. Conversely, since we expect constructions with more complex relation types, such as symmetric and asymmetric relations, to appear in later stages of language emergence, we predict that younger ISL signers will show more examples of these relations than older ISL signers. Assuming that relations across propositions are more complex than relations within propositions, we expect older signers to convey symmetric and asymmetric relations within propositions more often than across propositions, and younger signers are expected to mark more across proposition relations than older signers.

### Participants

Fifteen deaf ISL signers were filmed as part of this study. They represent three age groups, five in each: younger (18–29), middle (30–54), and older (55+ years). Participants ranged in age from 18 to 68 years (mean age: 42 years, 6:9 male: female), and their preferred language was ISL.

The oldest group of signers in our study have varied language backgrounds. Some were born outside of Israel, immigrating to Israel at a young age. Despite this, the first and most preferred language for the older signers in our dataset was ISL. The language of this group, like subsequent age groups, is by no means a home sign system. It qualifies fully as a language, since it has a large, conventionalized vocabulary and linguistic organization; it is the preferred language of its users; and it fulfills all communication and social functions of language throughout the larger community of 10,000 (See Meir and Sandler, 2008 for details). We did not control for heterogeneity (i.e., variation in terms of education and literacy), because it is this sort of variation that characterizes the language of this age group, and which was the model for the younger groups. Younger signers in our dataset are less heterogeneous than older signers, as they were exposed to peers and adult models from a young age, and attended school in deaf education frameworks.

All participants of all three age groups either grew up in deaf families (70% participants in the two younger groups) or have signed from a very young age, and all use sign language as their primary means of communication. This was confirmed by a detailed questionnaire containing information about language use throughout the lifetime of the participants. Consent was obtained from all participants for their involvement in the filming, and signers were compensated for their time. Filming took place at the University of Haifa Sign Language Research Lab.

### Task Procedure

Participants were asked to tell a personal life story to a deaf native signer research assistant of middle age. Narratives ranged in length from 3–40 min. We extracted 2 min from the middle of each narrative for this study (30 min of data in total). We did not analyze the beginnings of narratives as our aim was to analyze naturalistic signing, when the signer had become accustomed to the presence of the camera.

### Coding Units of Analysis and Discourse Relations

Narratives were divided into intonational phrases based on manual signals. Previous research has demonstrated that manual signals, such as pause, hold and reduplication, correspond to phrase-final lengthening in ISL, and are reliable signals of intonational phrase boundaries, often accompanied by blinks in ISL^5^ (Nespor and Sandler, 1999). The intonational phrase is the main domain of non-manual intonational contours. In other words, facial expression and head movements, corresponding to intonation, systematically align with intonational phrase boundaries (Nespor and Sandler, 1999; Dachkovsky et al., 2013). As in spoken languages (e.g., Chafe, 1984; Du Bois, 1985), intonational phrases can be considered as roughly corresponding to thought units. In this study the intonational phrase was the basic unit in the analysis of the distribution of non-manual signals marking discourse relations. All coding was completed using ELAN (Crasborn and Sloetjes, 2008), a video annotation software.

Each discourse relation was identified based on reliable markers in the sign language literature (see Table 2). The first relation, additive, is often described in the sign language literature as listing. It is expressed by a movement of the head, often in a thrusting action, along the forward-back axis, with or without movement of the torso. This has been noted in a number of sign languages (Wilbur and Patschke, 1998 in ASL; van der Kooij et al., 2006 in NGT; Sandler, 2012 in ABSL; Puupponen, 2017 in Finnish Sign Language). The second relation, the symmetric relation, is marked by opposite torso or head leans (van der Kooij et al., 2006 in NGT; Man, 2008 in Hong Kong Sign Language; Crasborn and van der Kooij, 2013; Puupponen, 2017 in Finnish Sign Language). A similar contrastive display has been noticed for ISL (Meir and Sandler, 2008).

**Table 2:** Marking for each relation in ISL.

Relation	Gloss of ISL example and translation	Marking in ISL	Example of ISL marking
Additive within proposition (no examples of additive across proposition)	[FATHER] [HAMMER] [SAW] [PAINT] *My father was hammering, sawing, and painting.*	Head thrusts	


			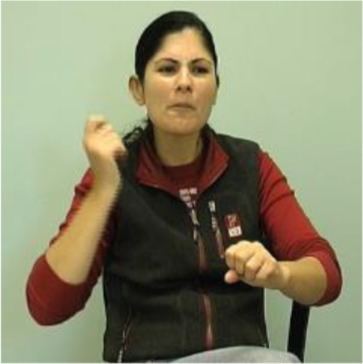
			HAMMER (head thrust)
Symmetric within proposition	[TEL-AVIV] [HAIFA] [COMPETE] *It was Tel Aviv vs. Haifa.*	Contrasting head and/or torso positions	


			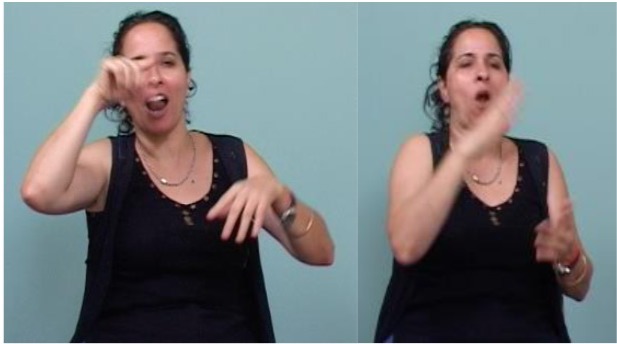
			TEL-AVIV (torso and head tilt right) HAIFA (torso and head tilt left)
Symmetric across proposition	[ALSO LANGUAGE SIGN-LANGUAGE] [ALSO ME NO RULE-OUT IMPORTANT ORAL] *As well as sign language (being important), I don’t rule out the importance of spoken language.*	Contrasting head and/or torso positions	


			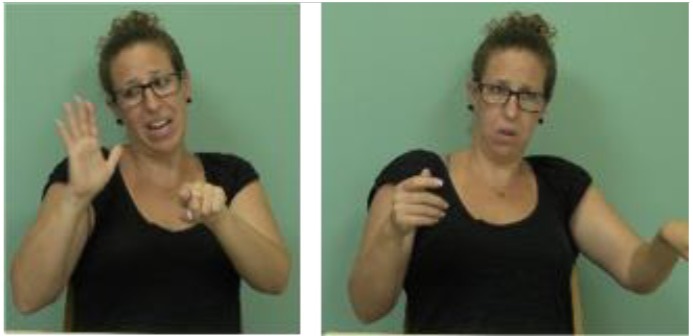
			ALSO (head tilt left) RULE-OUT (head tilt right)
Asymmetric within proposition	[TWO-OF-THEM WIFE FATHER] [TWO-OF-THEM STAY] *My father and his wife they both had to stay.*	Head forward, brow raise, and retraction	


			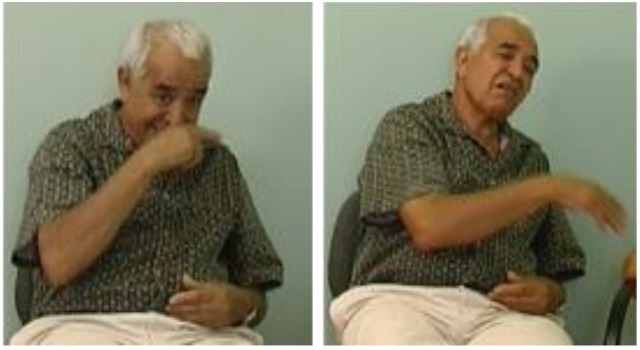
			FATHER (head forward, brow raise) STAY (retraction)
Asymmetric across proposition	[BECAUSE B-12 NO] [NEED INJECTION] *(Doctor said)…because you’re not getting vitamin B12, you need injections.*	Head forward, brow raise and retraction	


			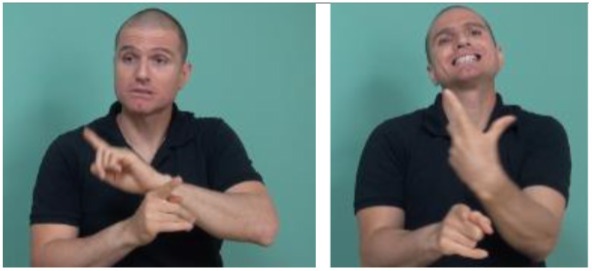
			NO (head forward, brow raise) INJECTION (retraction)

*The underlined words are the signs represented in the images*.

Finally, a number of articulations of the body have been associated with asymmetric relations. For example, raised brows has been shown to characterize subordinate constructions, such as conditionals and temporal clauses, as well as asymmetric relations within propositions such as between topic and comment (Liddell, 1980; Cecchetto et al., 2006; Dachkovsky and Sandler, 2009; Gökgöz, 2009; Fenlon, 2010). These relations are by definition asymmetrical because one constituent provides background information for the other constituent. Another marker, forward torso lean and/or forward head movement, marks asymmetric relations across the proposition, e.g., in conditional and relative clause constructions (Dachkovsky and Sandler, 2009; Dachkovsky et al., 2013; Dachkovsky, 2017). Forward head movement commonly combines with raised brows to mark dependent constructions (Nespor and Sandler, 1999; Dachkovsky and Sandler, 2009; Dachkovsky et al., 2013).

In total, for the present study we coded 17 articulations. They included movements of the torso (thrust, forward, back, tilt left, tilt right, turn left, and turn right), and head (thrust, forward, back, tilt left, tilt right, turn left, turn right, up, and down), as well as eyebrow raises. We made the distinction between head or torso thrusts or head and torso movements. The two share the same direction, forward and back, while a thrust has a quicker movement.

### Data Analysis

From here on, we use the term ‘marking’ to refer to the articulations of the body, which accompany a given construction. We are aware that this term is traditionally restricted to conventionalized grammatical units such as morphemes, but here we follow the sign language literature which has long studied ‘non-manual markers,’ and we use the term in a broader sense, to mean any articulatory action corresponding to a linguistic unit, agnostic with respect to the degree of conventionalization. Another caveat before we proceed: apart from brow raises, our coding did not include facial expressions^6^, and this awaits future research.

We investigate the frequency in the marking of three different types of relations: additive, symmetric, asymmetric. We follow these relations at two levels of discourse: within and across propositions. Together with discourse relations, we investigate three parameters that accompany the increase of language complexity, as described in **Measuring Relations and Complexity in Discourse**: (1) systematicity, (2) convergence on a particular marker, and (3) articulatory reduction of these markers, which is also an indication of their conventionalization. We measure (1) systematicity in terms of frequency of marking of these relations – we calculate the proportion of intonational phrases marked with each relation during 2 min of narrative and (2) convergence in terms of number of marker variants for the same function. Finally, we measure (3) the reduction of articulatory effort by (a) number of articulators and (b) type of articulators, where a decline in the number of articulators as well as a reduction in size of articulators is an indicator of a reduction in marking. For example, the head is a smaller articulator and its activation results in less muscular activity and displacement in space than the torso. Each specific marking (e.g., torso thrust) was calculated as a fraction of the total marked instances for each relation (e.g., 20% of additive relations marked by a torso thrust). The results of these analyses were further correlated with the complexity of discourse relations in order to see if the changes in the parameters of systematicity, convergence and reduction are indicative of an increase in complexity.

A total of 1473 tokens were analyzed. For every intonational phrase, we counted whether each relation was present, in order to make the tokens binary. For our study, we were interested in whether the frequency of each relation is predicted by the signer’s age. To this end, we included age as an independent and continuous variable (i.e., entering individual ages in years rather than categorical age groups). We conducted multivariate logistic regressions using a program known as Rbrul (Johnson, 2009) for each individual relation. Rbrul quantitatively evaluates the influence of multiple factors on variation (Rand and Sankoff, 1990). We included participant as a random effect in order to account for the effects of individual differences (Baayen, 2008; Jaeger, 2008).

## Results

### Relations per Intonational Phrase Unit

The total number of relations produced in our dataset was 755. On average, each signer produced 50 relations of varying degrees of complexity during 2 min of spontaneous narrative. We also found that on average signers across age groups produced similar numbers of intonational phrases during 2 min of narrative (*O* = 100, *M* = 95, *Y* = 100).

Table 3 presents the average percentage of all intonational phrases containing each relation, displayed by each age group. Some relations appear in similar distribution across all age groups. For example, additive constructions are present in 0.12 for younger, 0.13 for middle and 0.10 for older signers. Other constructions appear to be more frequent for some age groups than for others. For example, asymmetric relations across propositions (e.g., clausal dependencies) are more frequent in the narratives of younger signers (0.08) compared to older signers (0.01). We now present our statistical findings for types and levels of relation, leading us to an interpretation of the data relative to our hypotheses.

**Table 3:** Proportion of relations per intonational phrase.

Construction	Older	Middle	Younger	Total
Additive	0.098	0.13	0.118	0.346
Symmetric within proposition	0.018	0.037	0.042	0.097
Symmetric across propositions	0.03	0.061	0.108	0.199
Symmetry	0.146	0.228	0.268	0.642
Asymmetric within proposition	0.116	0.12	0.074	0.31
Asymmetric across propositions	0.018	0.04	0.076	0.134
Asymmetry	0.134	0.16	0.15	0.444
Total number of intonational phrases	500	475	500	1475

### Types of Relations

**FIGURE 4:**
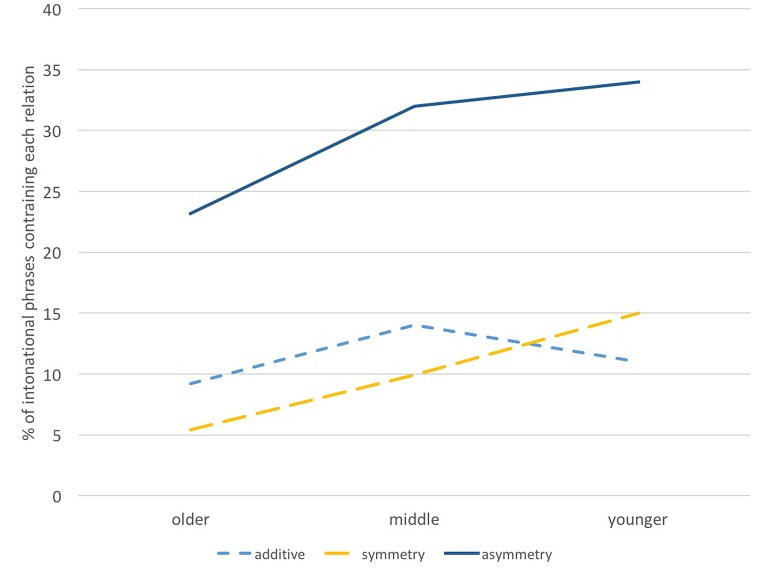
Percentage of intonational phrases containing each relation, displayed by age group.

We investigated age-related differences for additive, symmetric and asymmetric constructions, using statistical methods, to see whether the presence of a particular relation type is predictable by a signer’s age. The simple, additive type of construction occurred at the within-proposition level only, and was common across age groups. Multiple regression analysis reveals no significant differences with age for additive relations (*p* > 0.05). However, presence of symmetric constructions (log-odds^7^ -0.029, *p* = 0.015) and asymmetric constructions (log-odds -0.014, *p* = 0.0233) was significantly predicted by age. The results show that these relations are used significantly more by younger signers. The results are plotted as percentages in Figure 4 below.

### Levels of Relations

For levels of relations (within or across propositions), we investigated whether there were any age-related differences. Here we found a difference between the two levels. The data show similar numbers of symmetric and asymmetric relations within propositions across all age groups – there were no significant age-related results for symmetric or asymmetric relations within propositions. While no significant age differences were found for symmetric constructions across propositions (*p* > 0.05), we did observe a trend, such that younger signers mark symmetric constructions across propositions more than older signers (*Y* = 11%, >*M* = 6%, >*O* = 4%). We expect this result to be significant with the addition of more tokens.

For asymmetric constructions across propositions (e.g., main/subordinate constructions, i.e., embedding), age was found to be statistically significant. Multiple regression revealed that presence of these relations was predicted by age (log-odds -0.043, *p* < 0.01), with significantly more relations found in the signing of younger signers.

### Convergence in Marking of Relations

Our results point to increasing convergence; that is, there is less variation in the ways in which relations are marked in the younger signers. The findings for each relation are as follows:

(a) For the marking of *additive relations*, older signers mark these relations with a number of coexisting variants, with similar frequencies (torso thrust 30%, head and torso thrust 19%, head thrust 25%, etc.) Younger signers employ the same variants as the older signers, but they show clear convergence in the direction of one variant for the marking of additive relations: head thrust 41%.
(b) For *asymmetric relations within* the proposition, the main marking used by older signers is manual timing, i.e., phrase final lengthening (32%), followed by a number of other frequent variants (brow raise and forward head movement 18%, brow raise alone 15%, and forward head movement alone 12%). Younger signers, however, converge toward two main signals making up the majority of the marking (brow raise and forward head movement 35% or forward head movement alone 38%).
(c) While 44% of *asymmetric relations across* propositions are marked with a brow raise and forward head movement by older signers, the majority of the marking (56%) is randomly divided among other unrelated signals represented by single occurrences (e.g., head tilt, head turn, or brow lower). Younger signers again show more efficient and systematic use of the body, using only two major variants in equal distribution, one a reduced version of the other (brow raise and forward head movement 34% or forward head movement alone 34%).
(d) For *symmetric relations within* propositions, older signers use three distinct variants (torso and head tilt 50%, torso tilt 25%, and head tilt 25%) while all of the marking for younger signers is attributed to two variants, i.e., articulation by one articulator or the other (head tilt 53% and torso tilt 47%).
(e) For *symmetric relations across* propositions, we find no clear reduction in the number of variants used across age groups. That is, we do not see convergence on particular variants. Instead, we see a reduction in the number and type of articulators used (reported in **Changes in Number and Type of Articulators** below).

### Changes in Number and Type of Articulators

**FIGURE 5:**
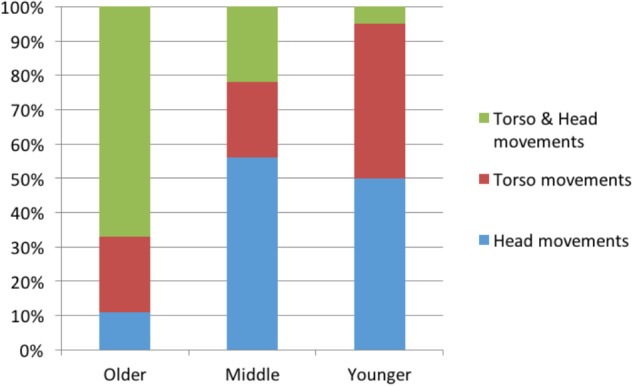
Decrease in multiple marking of symmetric relations within propositions.

Further analyses of the data reveals differences in the number and type of articulator activated. We find three main age differences; the marking of relations by younger signers is characterized by: (a) fewer articulators, (b) less use of the torso, and (c) composites are split into individual markers. We present the findings below:

(a) Fewer articulators – we collapsed our findings to consider the average number of articulators used to mark a discourse relation by signer age group (see Table 4). Younger signers in all relation types and levels are more likely to use a single articulator to mark a relation (64%) than older signers (39%). Older signers favor the use of two articulators in most cases.
(b) Less use of the torso – in additive relations and symmetric relations across propositions we see a reduction in the use of the torso across age groups, with younger signers using the torso less than older signers. Asymmetric relations, within and across propositions, rarely use the movement of the torso and therefore we do not see any change across the age groups for this parameter.
(c) Composites split into individual markers – in nearly all relations, older signers tend to use a composite of articulators simultaneously for the same relation (e.g., torso and head tilt simultaneously for symmetric within proposition). Younger signers, rather than simply moving toward the use of one dominant marker exclusively, split the composite into individual articulators and use either one of the articulations used in the composite (e.g., torso tilt or head tilt for symmetric within proposition). We discuss the implications of this trend in the **Discussion: Bodily Marking Emerges Gradually**. In Figure 5 below, we categorize the use of head and torso movements in symmetric relations and demonstrate the reduction in composites and the increase in individual head or torso use (composities: *O* = 67%>, *M* = 22%>, *Y* = 5%).

**Table 4:** Average percentage of articulators per discourse relation.

Age group	1 Articulator	2 Articulators	3 Articulators	Manual timing
Older	38.6%	45.2%	7.4%	6.6%
Middle	47.2%	45%	5.2%	2.6
Younger	63.8%	33.8%	2.4%	0%

**FIGURE 6:**
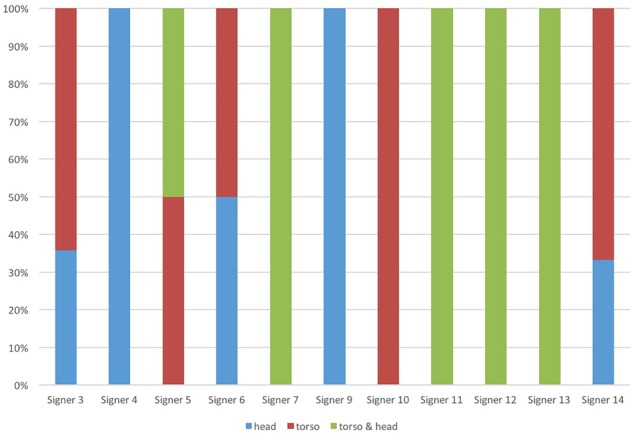
Variability of the marking of symmetric relations within proposition. Signer 1, 2, 8, and 15 showed no examples for this relation.

While the patterns across age groups are clear, we do find some variation at the level of individual signers, as shown in Figure 6. Despite this individual variation, results were significant at the age group level, a finding to which we return below, in **Convergence: Increased Systematicity of Bodily Marking**.

## Discussion: Bodily Marking Emerges Gradually

Our results suggest that a signer’s age – and according to our analysis, the age of the language – is an important predictor of frequency, systematicity, and complexity of relation marking. Frequency of marking varies depending on relation type and level. Our results indicate that younger signers mark more symmetric and asymmetric relations than older signers, despite the fact that all signers produced similar numbers of intonational phrases. There were no significant differences in the frequency of marking for simple additive relations, however. We also found a difference between the frequency of asymmetric relation marking depending on whether they were relations within or across propositions, with younger signers marking more asymmetric relations across propositions than older signers. Systematicity of marking differed across age groups. While older signers used larger articulators (the torso) and often redundantly marked relations with combinations of articulations, younger signers used the torso less, and they used fewer articulators, and often fewer composites, for a given relation. In the next sections, we discuss how our findings may be interpreted in terms of language emergence.

### Similarity in Number of Thought Units Across Age Groups

Interestingly, we found no difference in the numbers of intonational phrases produced across age groups. All signers produced roughly 100 intonational phrases during the 2-min narrative. Similar results were found for ABSL (Sandler et al., 2011). The number of signs in an intonational phrase and the internal complexity increased for younger signers, as did the speed of signing, but the number of intonational phrases was constant. In this study, we found that propositions produced by older signers consisted of fewer signs than propositions produced by younger signers. Based on the proposed correspondence between intonational phrases and thought units (Chafe, 1984; Du Bois, 1985), this finding suggests that humans generally conceive of and express thought units at the same rate, regardless of the internal linguistic complexity of each unit and of their interrelations.

This finding is important in verifying our results as it indicates that older signers are not simply marking fewer relations because they produce fewer or less complex thought units – instead, it strengthens our finding that older signers simply do not have the linguistic means for marking relations. The recruitment of different bodily articulators for different linguistic purposes takes time to emerge in a young sign language.

### Simple Adjacency Emerges Before Dependency

We suggest that those relations that are marked statistically more frequently by younger signers are undergoing change; that is, these relations are increasing as the language matures, while those used similarly across age groups are not undergoing change. In terms of language emergence, relations that are no longer undergoing change with age but show a stable use across all age groups (e.g., additive relations, see **Types of Relations**) may reflect earlier constructions that have stabilized in the language development process. Signals which are still undergoing change may reflect constructions that conventionalize later, that take time to emerge in a language. Therefore, since we find no age differences for additive relations, we propose that additive relations emerge before symmetric and asymmetric relations, and that relations within proposition (both symmetric and asymmetric) emerge before relations across propositions (i.e., symmetric and asymmetric across propositions). This supports our hypotheses, as schematized in Figure 3. Importantly, this shows that not all relations are marked from the outset of language emergence, and furthermore, that both the type and level of relation are important predictors in the ordering of emergence.

Since the relations that appear in the earliest stages of language emergence – additive type relations and relations within propositions – are less complex, we conclude that simple discourse relations emerge before more complex ones, as hypothesized. This directionality of language development – from additive relations to more dependent relations – has been attested in numerous studies in the framework of grammaticalization, in well-researched European languages, like Latin (Lehmann, 2008), and also in less studied languages, such as Saliba, an Oceanic language (Bril, 2007). Similar findings have been shown in the development from pidgins to creoles (e.g., Bruyn, 1995). However, since all of these studies involve old, well established languages or languages descending from them, conclusions could not be drawn regarding the emergence of a language from scratch. The current study fills this gap.

### Convergence: Increased Systematicity of Bodily Marking

In most types of relations that we analyzed, the marking becomes more convergent as the language matures. In other words, signers gradually converge on one or two signals to mark a specific relation from a larger number of signals. In doing so, the degree of variation decreases over time, with fewer coexisting variants observed in the signing of later generations (i.e., younger signers). In some cases, we see that signers in the older generation fail to explicitly mark the relation at all, only using manual signals to mark the intonational phrase boundary, or mark relations with a number of coexisting variants, with no indication of convergence among older signers toward a particular marker for each relation.

Increase in conventionalization has also been attested in an earlier study on the grammaticalization of relative clause constructions in ISL (Dachkovsky, 2017), as noted above. Similar processes have been attested in child language acquisition in a number of spoken languages (Berman, 1988, 1996; Givón, 2009b; Tomasello and Brooks, 2016). Results of experiments on language acquisition point in the same direction. For example, Hudson Kam and Newport (2009) investigated what learners acquired when their input contained inconsistent grammatical morphemes by manipulating the degree of input inconsistency and the age of the learners (children vs. adult). They demonstrate that only children, not adults, regularize inconsistent input and make their output less variable and more systematic. These results suggest that children may play a unique and important role in creole formation by regularizing grammatical patterns. And indeed, this is the trajectory of changes shown for pidgins and creoles (e.g., Muysken, 2001; McWhorter, 2005; Plag, 2008).

Literature on conventionalization suggests that the effect of regularization through repeated learning and use is amplified more when measured at the level of the whole population, rather than at the level of an individual language user (Smith and Wonnacott, 2010). The study reported here comes to exactly the same conclusions, but the evidence comes from a language in the visual modality. Specifically, we demonstrate that the convergence on specific articulators and increase in systematicity are a cumulative result of comparison across the age groups (see Figure 5 above), whereas individual signers display considerable variability in their signing, as shown in Figure 6.

### Reduction of Marking in Terms of Number and Type of Articulators

So far we have demonstrated that as the language matures there is an increase in the frequency and systematicity of marking which directly corresponds to the degree of complexity of these relations. In addition to this, our findings show a gradual reduction in the number and type of articulators, in line with grammaticalization studies in established languages, as outlined below.

#### Type of Articulators

**FIGURE 7:**
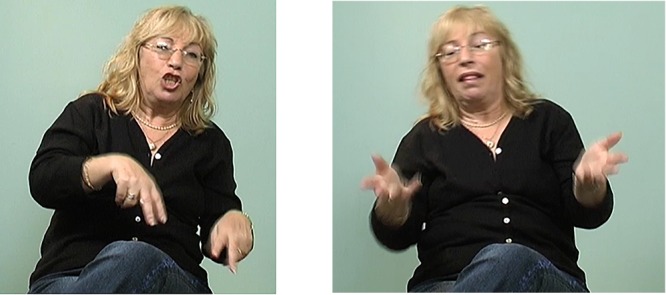
Marking of a symmetric relation by an older signer with opposite tilts of head and torso together. head and torso tilt left head and torso tilt right EXIST FOOD            NOT-EXIST FOOD *Sometimes there was* *food and sometimes there wasn’t food (The underlined words in the glosses above are the words signed in the corresponding figures.)*.

**FIGURE 8:**
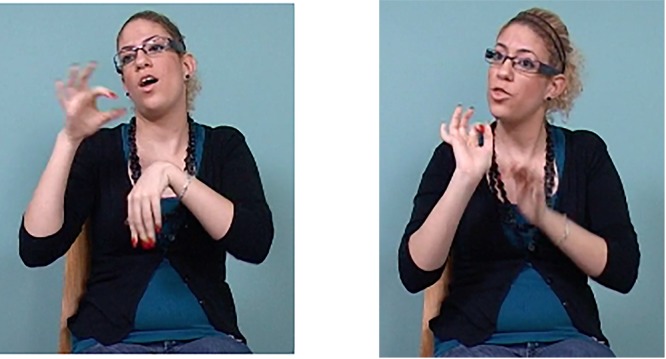
Marking of a symmetric relation by a younger signer with opposite head-only tilts. head tilt right head tilt left BEFORE DOCTOR HE REPLACE            DIFFERENT NEW DOCTOR SHE SIGN GOOD

In addition to the number of articulators, we also find a change in the type of articulator involved in marking. For symmetric relations, older signers typically displace the head and torso together (see Figure 7). Younger signers, however, in most cases engage only the head or only the torso in the marking of relations (see Figure 8). The former change, from head and torso to only head, shows that the use of a larger, grosser articulator (the torso) is replaced by a smaller, subtler articulator (the head).

A comparable change has been observed in the use of different arm joints – studies on sign language acquisition have found that signers change from first using joints closer to the body (e.g., movement of the shoulder) to using joints further from the body (e.g., movement of the elbow) (Lavoie and Villeneuve, 1999; Takkinen, 2003), causing reduction in the overall size of the sign. This also resembles findings by Mineiro et al. (2017), in which signing size decreased in the early stages of a new sign language. It may be that signers of a new language become gradually more efficient in the use of their articulators and that as a result signing becomes reduced as the signer is able to reduce their production effort. This seems to hold true in studies on young sign languages with later generations of signers using their bodies in a less holistic way compared to earlier generations in the marking of various linguistic functions (Kegl et al., 1999; Aronoff et al., 2003). Older signers in NSL and signers of ISL (compared to ASL) typically used their whole bodies for representing character viewpoint (i.e., overt constructed action, whole-body classifiers). While reduced effort might account for this replacement of larger articulators (e.g., torso) with smaller articulators (e.g., head), this does not explain why younger signers in ISL continue to use the torso as a relation marker, without moving the head. In the next section, we propose that signers benefit by separating the use of the torso and the head in order for one to be made available to participate in other linguistic functions.

#### Number of Articulators

**FIGURE 9:**
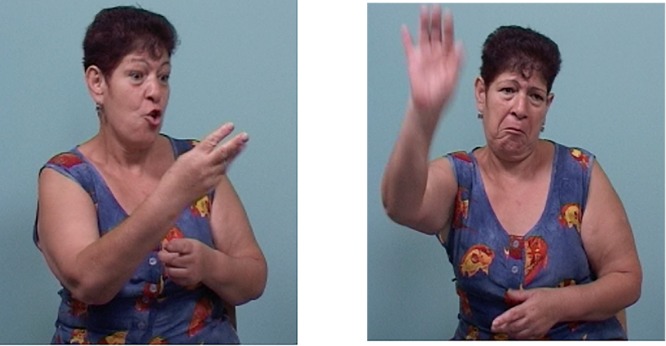
Marking of an asymmetric relation across propositions shown by an older signer – torso shift, forward head movement, head turn, and eyebrow raise accompanies the first part of this construction. torso and head turn, brow raise, head forward head tilt left FATHER SAY YOU TWO THREE            ME ONE GO *My father said that if there had been two or three of us (girls), then I could have gone*.

**FIGURE 10:**
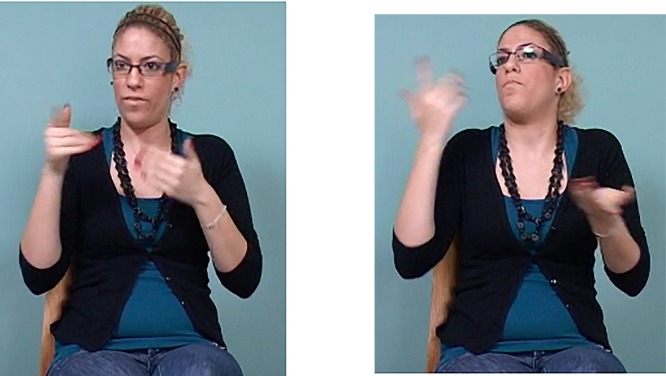
Marking of an asymmetric relation across propositions shown by a younger signer – forward head movement accompanies the first proposition *When my mother needed urgent treatment* and backward head movement accompanies the second proposition, *we did not know what to do*. head forward head back SHE MOTHER EMERGENCY PAST SHE            WE THINK WHAT-TO-DO *When my mother needed urgent treatment we did not know what to do*.

Generally, we see a decrease in the number of articulators when we compare across the different age groups. Older signers tend to use multiple articulators – for example, the signer in Figure 9 moves her head forward, raises her eyebrows and turns her head, compared to younger signers who use a single articulator for the marking of asymmetric relations, such as dependencies (typically, subordination, see Figure 10) – for example, with only forward head movement. This change across age groups reflects a diachronic change, such that there is a decrease in the number of articulators used for the marking of these discourse relations as the language matures.

### Less Is More: Implications for Compositionality

In later stages of the language, signers use fewer articulators to mark a single linguistic function. What we see here is a reduction in redundancy of articulator marking as the language evolves. During language emergence one might expect that redundancy of feature marking may increase in order to improve comprehensibility (e.g., Pinker and Jackendoff, 2005; Bazzanella, 2011). That said, languages must find a balance between comprehension and economy (Zipf, 1949). ISL at only 90 years of age is considered to be a young sign language^8^, and yet studies of its development have shown that it has changed dramatically relative to other sign languages of a similar age in villages and towns in Israel (Padden et al., 2010a; Meir and Sandler, in press). This has been attributed to the size and heterogeneity of the ISL population, as well as to its use in a range of different domains including education, interpreting programs and the media (Meir et al., 2012). As a result, it is perhaps unsurprising that we see a reduction in redundancy of features in this language, as we find in spoken languages also (e.g., Jaeger, 2010; Bazzanella, 2011). Considering the mixed backgrounds of older deaf signers in our dataset, we might expect to find clearer patterns of reduction in older signers of ABSL, where the sign language community receives relatively little contact from other languages, spoken or signed.

With decreased redundancy and increased systematicity come a number of advantages. In the case of sign languages, by using one fewer articulator in the marking of a specific function, the signer is able to recruit that articulator to mark a different function (Sandler, 2012, 2013 for ABSL). Simultaneous markings of different functions by different articulators do not stand out in our dataset, which we attribute in part to the fact that we restricted our analysis to markers of a subset of discourse relations. For example, markers of information status, indicated by facial expressions (Dachkovsky et al., 2013) and independent actions of the non-dominant hand (Liddell, 2003; Sandler, 2012, under review), were not included in the analysis. We predict that the inclusion of such structures in future research will show that increased linguistic complexity is reflected by increased simultaneous activation of different articulators for different functions, as demonstrated for ABSL.

**FIGURE 11:**
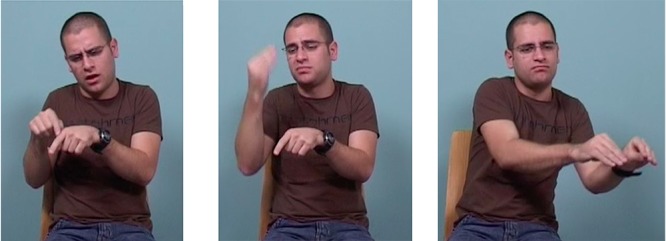
Mapping between simultaneous discourse relations and simultaneous articulations. Opposite torso tilts signal the symmetric contrast between the two major constituents (two different schooling situations); the non-dominant hand (=nd) marks topic continuity; and forward-backward head movement marks the asymmetric relation between dependent and matrix clauses within the first coordinated constituent. head forward    head back ____________  ______________________________________________________ torso tilt right                                       torso tilt left _____________________________________________________________________ _____________ [[ME GROW-UP SCHOOL THERE] [END SCHOOL THERE CLOSE DEAF INTEGRATE NO-MORE] [MOVE-HERE]] [[When I was at that school,] [they closed the deaf program]], [and I moved to another school.]]

However, in the present study, there are some examples of such simultaneous complexity in young signers. For example, one young signer produced an utterance containing two relations (see Figure 11 above). The utterance can be translated as:

*[[[When I was at that school,] [they closed the deaf program]], [and I moved to another school*.*]]*

In this example, the signer marks a symmetric (coordinate) contrastive relation between the two main constituents by tilting his torso to the right for ‘*When I was at that school, they closed the deaf program*,’ and to the left for ‘*and* *I moved to another school.*’ The first constituent has as its topic ’*that school,’* and topic continuity is marked by keeping the non-dominant hand (‘nd’ – indexing the location of the school) in the signing space throughout.

The information provided in the first constituent is further subdivided into two clauses in an asymmetrical (dependent) relation to one another (‘*When I was at that school*’ and ‘*they closed the deaf program*’). Here the signer moves his head forward for the dependent clause, ‘*When I was at that school*,’ and back for the matrix clause, *‘they closed the deaf program’* – while keeping the body position constant throughout this whole complex first constituent, and changing it only for the second constituent, ‘*I moved to another school.’*

By separating out articulators for different functions, two discourse relations can be conveyed simultaneously – symmetry by torso tilt and asymmetry by head movement – so that the compositionality of the discourse relations is reflected in the compositionality of bodily articulations.

## Conclusion

Differences between the frequency of occurrence and the type and consistency of marking of discourse relations by younger and older signers reveal specifically how this young language becomes increasingly complex over time. The most striking finding in this regard is that the asymmetrical relations across propositions – that is, typically, subordination – are significantly more common in the younger than in the older signers. This finding is commensurate with a more limited study on ABSL, where dependent, subordinate structures were found only in younger second generation signers (Sandler et al., 2011).

We also find here that the marking of the discourse relations becomes more systematic over time, in that they become more reliably marked by the same articulators. As the language matures, the signals become more specialized, with fewer articulators dedicated to a particular function, and with finer movements, involving the head more than – and separately from – the torso. Systematic use of the body for linguistic organization mirrors the emergence of linguistic complexity.

The overall picture painted by these results is that of a system that begins with simple relations, unconstrained, redundant form, and high variability. Thus, while the system of older signers clearly has linguistic properties, as we have explained, the aspects of its organization uncovered here are less systematic. We see gradual change in all of these parameters, resulting in a more conventionalized, systematic, constrained, and compositional sign language.

The previous work on ABSL has suggested that the markers of different discourse functions do not appear all at once. The present study on ISL expands and enriches that proposal by demonstrating that the recruitment of the specific bodily articulators follows a rule-governed functional trajectory – from less complex to more complex discourse functions. Another hypothesis put forward by the earlier study, that the earlier stages of a sign language are characterized by a more holistic use of the body, was also supported in the present study. The current study suggests benefits that the specification of the articulators might contribute in the process of language emergence. Moreover, the findings here shed light on the complex tug of war between conventionalization and regularization on the one hand and variability and diversity on the other (see Meir and Sandler, in press). A lower number of participants in the previous studies might have obscured that issue. In addition, until now, little has been reported at the level of discourse about the path from a system with idiosyncratic characteristics to a more constrained and complex sign language. The work we report here reveals steps along that path.

## Ethics Statement

All participants provided written informed consent to participate in this study. The study was approved by the Ethics Committee/IRB of the University of Haifa (https://resau.haifa.ac.il/index.php/en/policy-requirement/ethics-committee-institutional-review-board-irb).

## Author Contributions

SD is the lead author. RS and SD completed the data coding and analysis. RS contributed toward writing the paper. WS is the principal investigator on the project and contributed toward the writing and interpreting of the results.

## Conflict of Interest Statement

The authors declare that the research was conducted in the absence of any commercial or financial relationships that could be construed as a potential conflict of interest.

## Footnotes

2 Discourse segments can be characterized as formally distinct units of discourse.

3 Additive relations are also known as iterative (Givón, 2009b).

4 Sociolinguistic factors can affect the degree of language complexity in young and emerging languages. This is mostly as a result of external factors, such as the degree of contact with other languages, the size and social structure of the language population, and the number of second language users (Bolender, 2007; McWhorter, 2007; Wray and Grace, 2007; Lupyan and Dale, 2010; Meir et al., 2012; Trudgill, 2012; Meir and Sandler, in press in signed and spoken language research). The comparison of ISL with other languages using the same criteria is a topic for future research.

5 Eyeblinks were also found to align with intonational phrase boundaries by Wilbur (1994).

6 For a study of the emergence of a particular structure in ISL that explicitly incorporated facial expression, see Dachkovsky (2017).

7 Log odds measure the strength of the relationship between a factor (in this case, age) and a dependent variable (in this case, presence of a relation type in each intonational phrase). Results with positive log odds, above 0, indicate a positive correlation and results with negative log-odds, below 0, indicate a negative correlation between the variables - the higher the value the stronger the correlation.

8 In comparison with British Sign Language, at 300 years old, which is considered to be old among established sign languages (Kyle and Woll, 1985).
